# Amniotic Membrane: An Approach to Periodontal Regeneration

**DOI:** 10.7759/cureus.27832

**Published:** 2022-08-09

**Authors:** Eileen J Law, Haslina Taib, Zurairah Berahim

**Affiliations:** 1 Dental Clinic, School of Dental Sciences, Health Campus, Universiti Sains Malaysia, Kota Bharu, MYS; 2 Periodontics Unit, School of Dental Sciences, Health Campus, Universiti Sains Malaysia, Kota Bharu, MYS

**Keywords:** periodontal healing, regenerative medicine, tissue engineering, periodontal regeneration, amniotic membrane

## Abstract

Over the years, various materials have been used for scaffold-based periodontal tissue engineering to regenerate lost periodontal tissues. The use of amniotic membrane (AM) as a scaffold for periodontal regeneration has gained great interest among researchers. This narrative review aims to appraise the properties of AM and its potential clinical applications in periodontal regeneration. PubMed, ScienceDirect, Scopus, and Wiley Online Library databases were searched for relevant articles that highlighted the properties and applications of AM in periodontal regeneration. AM has a unique structure and components contributing to its exceptional properties such as anti-inflammatory (presence of anti-inflammatory factors), low immunogenicity (presence of human leukocyte antigen-G), anti-scarring (downregulation of transforming growth factor-β), antimicrobial (expression of antimicrobial factors), promotion of epithelialization (production of growth factors), and reduction of pain (protection of exposed nerve endings). Its use in the treatment of periodontal tissue defect has shown to be effective. AM showed various beneficial properties as an ideal scaffold. Future studies and long-term clinical trials on the efficacy and survival rate of AM are required to completely understand the potential application of AM in periodontal regeneration.

## Introduction and background

Periodontal disease is a major public health issue that distributes globally and comprises a wide spectrum of conditions ranging from mild gingivitis to severe periodontitis [1,2]. According to the Global Burden of Disease Study 2016, severe periodontal disease was the 11th most prevalent condition in the world, with its prevalence ranging from 20% to 50% [3,4]. It is a chronic inflammatory condition initiated by bacteria in dental biofilm in the susceptible host, which can be modified by the presence of risk factors. In general, it can be classified as gingivitis and periodontitis.

The ultimate goals of periodontal therapy include the arrest of periodontal disease progression and complete reconstitution of all periodontal attachment to their original architecture and function that replicates its pre-disease structure [5]. Periodontal regeneration is defined as the restoration and reconstruction of the lost periodontium or supporting structures including the alveolar bone, cementum, periodontal ligament, and gingiva [6]. However, current conventional periodontal therapies show a limited potential for complete periodontal regeneration. Over the years, various methods have been used in achieving periodontal regeneration. The most common is guided tissue regeneration (GTR), whereby the membrane or other biomaterials are used as a barrier or scaffold in order to allow the desired cell to repopulate the periodontal defect area. The membranes that have been used include natural and synthetic biomaterials [7].

The amniotic membrane (AM) is the innermost layer of the fetal membranes, which is avascular and forms an amniotic fluid-filled sac that surrounds and protects the embryo. AM is translucent and is one of the thinnest membranes (approximately 0.02-0.5 mm) in the human body. It is made up of three distinct layers: (1) epithelium, (2) basement membrane, and (3) stromal matrix. The stromal matrix can be further divided into three layers, which are the inner acellular compact layer, the middle loose fibroblast layer, and the outermost spongy layer [8,9]. AM is routinely discarded post-partum. It is obtained after normal or cesarean deliveries under informed consent, which usually poses little to no ethical concerns. Consequently, it is a readily available and cost-effective biomaterial for scaffolds in tissue engineering [10]. Scanning electron microscopy analysis of AM revealed rough surface architecture with the presence of microporosity, which may provide a suitable platform for cell attachment [11]. AM was used for wound treatment more than a century ago as a skin graft substitute for open wound for treating burnt and ulcerated skin surfaces by which it can accelerate epithelialization and reduce pain [12-14]. In 1940, de Rötth [15] first reported the use of fresh amnion and chorion in ophthalmology to reconstruct the ocular surface in patients with symblepharon [16,17]. Since it was discovered that AM could be separated, sterilized, and safely used, amnion-derived cells have attracted much attention in dentistry, particularly for the regeneration of periodontal tissues [18].

From an updated review of the top five clinical applications of AM in regenerative medicine from 2015 to 2020, it was revealed that dermatology (specifically wound healing), orthopedics, ophthalmology, dentistry, urology, oncology, and otolaryngology used AM more compared to other specialties. AM only accounted for 6% in dentistry as compared to 32% in dermatology and 26% in orthopedics [19]. However, AM is one of the biomaterials that became an area of interest in periodontal application. The reports on its use in the management of gingival recession, furcation, and intrabony defects have shown positive outcomes [20]. Hence, due to the increasing number of studies in the field of regenerative medicine, studies are still needed to clarify the future prospect of AM in dentistry particularly periodontology [8].

Therefore, the purpose of this review is to appraise the properties of AM and its potential clinical applications in the field of regenerative periodontology.

## Review

Article search

A web search of all relevant literature was performed on the databases such as PubMed, ScienceDirect, Scopus, and Wiley Online Library. The following keywords were searched alone or in different combinations in the titles and abstracts: “amniotic membrane”, “periodontal regeneration”, “periodontal surgery”, “tissue engineering”, “regenerative medicine”. Relevant articles were identified, and duplicates were removed. Full texts of the identified articles that met the inclusion criteria were acquired and assessed. Articles were searched and retrieved from the reference lists of the initially selected articles for additional relevant studies. Searches were limited to articles in the English language and published from January 2001 until December 2021. The inclusion criteria for articles include clinical trials, case reports, and case series. The findings from the search are presented as a narrative review.

Results

**Figure 1:**
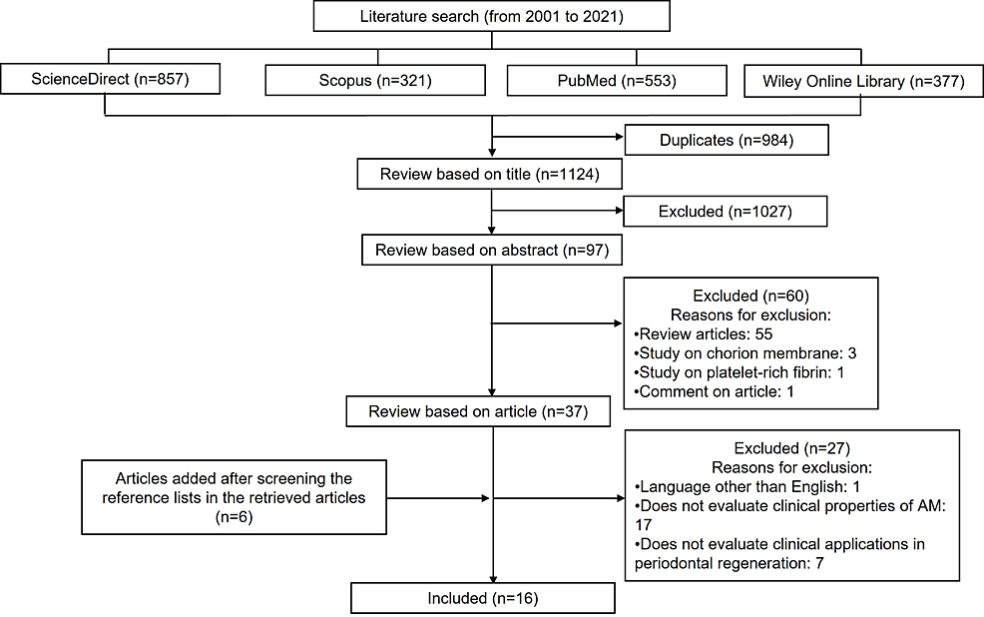
Flowchart of the literature search and selection process

During the initial search process, overall, 2,108 articles were found from the databases ScienceDirect, Scopus, PubMed, and Wiley Online Library. However, after further screening, which included removing duplicates and reviewing publications based on titles, abstracts, and articles, only 16 articles published in the year 2014 to 2021 were identified and included. Figure 1 depicts the process of conducting a literature search and the number of articles found.

Of the 16 articles, nine (56.25%) were studies that originated from India. Two (12.5%) were studies that originated from Iran. Other study origins were from Japan, Taiwan, Malaysia, Italy, United States of America, each (6.25%) respectively. Out of 16 articles, six (37.5%) were case reports, five (31.25%) were randomized controlled clinical trials, three (18.75%) were animal studies using a rat model, and one (6.25%) was an in vitro study and case series, respectively.

It was found that AM possesses many beneficial properties, which include antifibrotic, anti-inflammatory, antimicrobial, anti-scarring, mechanical strength, and flexibility [21]. One of the most widely reported properties of AM is its ability to reduce inflammation [22-25]. AM is also proven to have other properties such as low immunogenicity, pain reduction and promotion of epithelialization, self-adhesive, and aesthetics [26-28].

In regard to AM as a potential scaffold for periodontal regeneration, nine studies reported promising outcomes in the root coverage procedures for the treatment of gingival recession defects. Seven studies on GTR demonstrated that AM was an effective barrier able to enhance bone fill and improve periodontal parameters (probing pocket depth [PPD] and clinical attachment loss [CAL]). One laboratory study reported AM as a suitable scaffold for periodontal fibroblast cell growth [11]. The findings from all selected studies are summarized in Table 1.

**Table 1:** Detailed summary of the selected studies AM, amniotic membrane; PPD, probing pocket depth; CAL, clinical attachment loss; HPDLFs, human periodontal ligament fibroblasts

References	Study Origin	Type of Study	Properties	Clinical Applications of AM in Periodontal Regeneration
[26]	India	Case report	Self-adhesive, promotion of epithelialization, low immunogenicity, easily available, cost-effective	Stable and full root coverage of a Miller class I gingival recession defect seven months post-surgery
[21]	Japan	In vivo (rat model)	Antifibrotic, anti-inflammatory, antimicrobial, anti-scarring, mechanical strength, flexibility	AM acts as a scaffold for periodontal ligament stem cells to enhance periodontal regeneration and showed a monolayer of the cells on the amnion surface
[22]	India	Case report	Non-immunogenic, anti-inflammatory, antibacterial, reduction of pain, aesthetics	AM allograft in conjunction with gingival flap showed a complete root coverage of a Miller class II gingival recession with improved tissue architecture six months post-surgery
[27]	India	Case report	Lack of immunogenicity, antibacterial, reduction of pain, aesthetics	AM can be used as an allograft material in the treatment of root coverage to gain attachment level and reduce the length of the recession
[23]	India	Randomized controlled clinical trial	Anti-inflammatory, anti-infective, antimicrobial	AM functions as a barrier for guided tissue regeneration to increase bone fill and reduce PPD and CAL
[18]	India	Case series	Self-adhesive	AM as an autograft tissue in the treatment of shallow-to-moderate Miller’s class I and II recession defects showed a significant improvement in the clinical attachment level and width of keratinized gingiva six months postoperatively
[28]	Iran	Randomized controlled clinical trial	Self-adhesive, aesthetics	Coronally advanced flap with AM in the treatment of Miller’s class I and II gingival recessions decrease surgical operation time and patient discomfort
[24]	Taiwan	In vivo (rat model)	Anti-inflammatory, anti-angiogenesis, immunosuppression	AM and adipose-derived stem cell co-culture system increases bone regeneration in a periodontal osseous defect rat model by forming more hard tissues and showing better defect recovery
[29]	India	Case report	Promotion of epithelialization, anti-scarring, lack of immunogenicity, antimicrobial, antibacterial	AM can be used as an effective barrier in conjunction with bone grafts to treat an intrabony defect
[30]	India	Randomized control clinical trial	Promotion of epithelialization, anti-scarring, lack of immunogenicity, self-adhesive	Coronally advanced flap using AM showed a favorable outcome of root coverage percentage in the treatment of localized gingival recession defects by maintaining the structural and anatomical configuration of the regenerated tissues
[31]	India	Case report	Excellent handling properties, self-adhesive, easily available, uniform thickness	The combined approach of the coronally advanced flap and AM in the treatment of multiple adjacent gingival recessions showed significant root coverage and an increase in thickness of keratinized gingiva
[11]	Malaysia	In vitro	Biocompatible for cell growth, porous surface	AM serves as a scaffold for the attachment and proliferation of HPDLFs in periodontal tissue engineering
[32]	Italy	Case report	Promotion of epithelialization, reduction of pain, anti-scarring	AM acts as an allograft in the treatment of gingival recession in conjunction with coronally advanced flap and can promote palatal wound healing
[25]	Iran	Randomized controlled clinical trial	Anti-inflammatory, reduction of pain, anti-scarring, aesthetics	AM as a biological dressing on wound healing after free gingival graft surgery can prevent postoperative complications and help to accelerate healing
[33]	United States of America	In vivo (rat model)	Neovascularization, promotion of osteoconduction	Periodontal regeneration was enhanced in surgically created rat periodontal furcation defects by preserving its structure during cultivation and healing periods, supporting cell attachment and bone deposition
[34]	India	Randomized controlled clinical trial	Promotion of epithelialization, reduction of pain	AM as a barrier with biphasic calcium phosphate provides a better outcome in the management of periodontal intrabony defects by reducing PPD and CAL in chronic periodontitis patients

Properties of AM

Anti-inflammatory

Out of the 16 studies, five (31.25%) showed that AM has anti-inflammatory properties. Kumar et al. conducted a randomized controlled clinical trial to investigate the anti-inflammatory, anti-infective, and therapeutic effects of AM when utilized for GTR in confined interdental lesions [23]. The interleukin (IL)-1β and human beta-defensins (hBD)-2 levels were measured in the gingival crevicular fluid (GCF) of the test site (AM with bone graft) and control site (bone graft only). GCF is an inflammatory exudate that can be used as a non-invasive method to evaluate periodontal inflammatory reactions in a variety of clinical settings [35]. AM demonstrated a significant reduction in IL-1β level and an insignificant increase in hBD-2 expression in GCF. Increased hBD-2 levels play an important role in defense from periodontopathogens in human gingival tissues. The significant reduction of GCF IL-1β levels in AM-treated sites indicated that AM has a significant anti-inflammatory effect on periodontal tissues [23]. This finding is consistent with that of Kadkhoda et al. [25], whereby the inflammation was used as an objective measure of clinical healing. At all follow-up visits, the inflammation on the palatal donor site was more prominent in the control group, although the difference was significant only after 14 days post-surgery. On day 21 post-surgery, the inflammation score in AM group was “0,” which indicates no inflammation [25].

Several studies have shown that the incorporation of AM into collagen scaffolds enhanced its anti-inflammatory properties through chemical and mechanical effects. Chemically, there is a presence of various anti-inflammatory factors and substances such as activin A, IL-1 and IL-2 receptor antagonists, IL-10, endostatin, and tissue inhibitors of metalloproteinase (TIMP)-1, TIMP-2, TIMP-3, and TIMP-4, which inhibit endothelial cell proliferation, angiogenesis, and tumor growth [13,20,36,37]. Secretory leukocyte proteinase inhibitor (SLPI) and elafin have both anti-inflammatory and anti-microbial effects [8]. Chemical-mediated anti-inflammatory effect is also driven by the suppression of pro-inflammatory cytokines IL-1α, IL-1β, IL-2, IL-8, interferon-γ, tumor necrosis factor (TNF)-β, basic fibroblast growth factor, and platelet-derived growth factor [16]. Other than that, there is a decreased recruitment of inflammatory cells such as polymorphonuclear cells, CD3 cells, CD4 T cells, and CD11b cells [38,39]. In addition to the chemically mediated anti-inflammatory effect, the mechanical effect was demonstrated by AM, which serves as a physical barrier that confines inflammatory cells to the affected area and decreases inflammatory mediators. AM stromal matrix entraps T lymphocytes and results in apoptosis of the inflammatory cells [39]. Therefore, AM has considered being a suitable allotransplantation tissue due to its anti-inflammatory effect.

Low Immunogenicity and Immunomodulatory

Six of the (37.55%) 16 studies reported that AM has low immunogenicity and immunomodulatory properties. Rehan et al. studied the effectiveness of coronally advanced flap (CAF) with AM in the treatment of localized gingival recession defects [30]. The results were reported to be stable even after 18 months postoperatively, suggesting that AM forms a physiologic seal with the host tissue hindering bacterial contamination while supporting AM’s ability to decrease host immunologic response through localized suppression of polymorphonuclear cell migration. This finding is in accordance with the case reports of Shah et al. [22] and Shetty et al. [26] who reported stable results in AM-treated sites for six and seven months, respectively, post-treatment without recurrence of recession. The results from these reports are encouraging and demonstrated that amnion allograft is well-tolerated by the gingival tissues without any sign of immununorejection. In fact, immunosuppression is mandatory in skin allografts. However, AM transplantation for skin or corneal defects performed an exceptional lack of immunogenicity property by showing no signs of rejection in the absence of immunosuppression. This phenomenon result was most likely from the combination of anti-inflammatory, low immunogenicity, and immunomodulatory properties [40]. Low immunogenicity is important to create a biocompatible scaffold for tissue engineering.

The occurrence of acute rejection after transplantation of AM is very rare due to the fact that amniotic epithelial cells do not express human leukocyte antigen (HLA)-A, HLA-B, HLA-D, and HLA-DR antigens. Instead, amniotic epithelial cells express immunoregulatory factors HLA-G and Fas ligand on their surfaces. The expression of HLA-G is the main factor that prevents the rejection of the trophoblast because it is involved in the induction of immune tolerance by acting as a ligand for inhibitory receptors that present on the natural killer (NK) cells and macrophages [8]. The presence of interferon-𝛾 and other immunologic factors has been observed in the AM [9,20,41]. The immunologic factors secreted by the epithelial cells reduce the host immunologic response to prevent a maternal immune attack [18,23,42]. It was reported that there was no immunorejection observed from the transplantation of allogeneic periodontal ligament stem cell (PDLSC)-transferred amnion into swine periodontal defect models. No enhancement of T-cell and B-cell proliferation and immunoglobulin production was shown, thus suggesting the possibility of periodontal regeneration using allogeneic PDLSC-transferred amnion [21]. AM is also said to be immunomodulatory due to its unique molecular arrangement, which makes it invulnerable to maternal immune system responses. The cellular components of AM have active suppression activity on the immune cells’ activity through a strong paracrine secretion. This suggests that AM may have an immunomodulatory effect after transplantation, preventing the cellular cargo from being rejected [43]. Due to its success to prevent an allogenic or xenogenic immunologic reaction, AM has gained great interest in transplantation and tissue engineering. Despite these promising results, questions remain on the long-term efficacy and stability of AM as an immunomodulatory biological dressing. Therefore, to establish the efficacy and stability of AM, more randomized controlled clinical trials involving immunological investigations with longer follow-up visits are required.

Antimicrobial

The results of three studies (18.75%) showed that AM has antimicrobial properties. The study by Kumar et al. [23] reported that there was a minimal insignificant increase in the hBD-2 levels in sites treated with AM. This relatively small rise in the hBD-2 levels was caused by a significant reduction in the IL-1β levels. AM demonstrates an antimicrobial effect due to hBD production and by forming a biological “seal” with the host tissues, thus acting as a physical barrier against the outer environment. Defensins help in tissue proliferation, and the production of antimicrobial peptides by AM may promote periodontal regeneration [23]. It was suggested that the mechanism of antimicrobial action of AM is due to its role as a biological barrier against bacterial infiltration by closely adhering to the wound surface and preventing dead space formation and serous charge accumulation [37].

Other literature further explained that AM forms a barrier with the wound surface via fibrin and elastin linkages. This firm adherence helps in restoring lymphatic integrity, protecting circulating phagocytes from exposure, and allowing faster removal of surface debris and bacteria from the wound surface. There are two mechanisms mediating the antimicrobial activity: (1) direct, via secretion of antimicrobial factors such as human cathelicidin (LL-37), and (2) indirect, via secretion of immunomodulatory factors, which upregulate bactericidal activity and phagocytosis by immune cells. AM is also found to contain many bactericidal products of purine metabolism and lysozyme. A major group of antimicrobial peptides found in the AM is formed by defensins, mostly β3-defensin, that helps the epithelial surfaces to resist microbial colonization [38]. Apart from that, antimicrobial compounds found in amniotic cells, such as SLPI and elafin, act as components of the innate immune system to guard against infection. Treatment of AM with IL-1 receptor antagonist or lactoferrin also showed an antimicrobial effect [8]. Therefore, the antimicrobial property of AM has made it a suitable option for post-surgery applications in wound healing, burns, dental injuries, and ophthalmology because bacterial infection and biofilm growth are common in these sites [44].

Promotion of Epithelialization and Reduction of Pain

A case report described Miller’s class III gingival recession treated with a palatal epithelial-connective tissue autograft and AM. It was reported that surgical treatment with palatal epithelial-connective tissue graft and AM can help accelerate the epithelialization of the wound at the palatal donor site, reducing morbidity. A positive resolution of the treated recession, absence of infection, and complete reepithelialization of the palate treated with AM were observed 30 days post-surgery [32]. AM may act as a basement membrane that promotes epithelialization by aiding epithelial cell migration, basal cell adhesion, epithelial differentiation, and epithelial apoptosis prevention. AM also produces growth factors that stimulate epithelialization and have a pain-reducing effect [7,20,37]. It reduces inflammation and hydrates the wound bed, thus promoting faster healing. This membrane was also proven to promote rapid epithelialization of the palatal donor site wound with a reduction of post-operational pain, thus leading to less discomfort experienced by the patient [32]. AM promotes healing and wound epithelialization while reducing granulation tissue formation in large open wounds without any adverse reaction, as reflected by decreasing analgesics intake and pain scores as well as minimal discomfort postoperatively [25,27]. These results may be explained by the fact that the stromal surface closely adheres to the wound surface, and therefore the mucoid lining can protect the exposed free nerve endings in the wound area from external irritants and reduce pain sensation by preventing trauma and nerve stimuli [38,41]. Another mechanism proposed is AM causes downregulating of the pro-inflammatory cytokines, such as TNF-α and IL-6, and activation of neutrophils and M1 and M2 macrophages, which help to relieve pain [19].

Anti-Scarring

Scar tissue formation is a common occurrence during wound healing. Scar development is a complex biological process involving cell-cell and cell-matrix interactions driven by cytokines [45]. Kumar et al. [29] demonstrated the anti-scarring property of AM when used in conjunction with bone grafts to treat an intrabony defect. AM improves the overall regeneration due to its rich source of pluripotent stem cells, specialized proteins, and cytokines, thus promoting wound healing and reducing postoperative scarring [29]. In an 18-month clinical study to compare the efficacy of CAF using AM and platelet-rich fibrin (PRF) membrane in gingival recession, it has been demonstrated that CAF with AM is effective and showed better results than PRF membrane in providing clinically significant outcomes of root coverage by maintaining the structural and anatomical configuration of the regenerated tissues and enhancing healing through reduction of postoperative scarring [30].

This could be through secretion of vascular endothelial growth factor and hepatocytes growth factor that establishes a balance between transforming growth factors (TGF)-1 and TGF-2. Furthermore, there is a downregulation of TGF-β signaling modulated by hyaluronic acid, which suppresses the expression of TGF-β receptors such as TGF-β1, -β2, -β3 isoforms, and TGF-β type II receptor, inhibiting fibroblasts proliferation [18]. Differentiation of fibroblasts into myofibroblasts is also inhibited, thus reducing scarring [16]. Other contributing factor includes the reduction of protease activity due to the secretion of TIMPs [44].

Self-Adhesive

Five (31.25%) of the 16 studies have demonstrated that AM has a self-adhesive property. AM is able to adhere to the recipient's exposed root and proximal site upon placement on gingival recessions, thus eliminating the need for suturing [28]. AM can self-adhere and intimately adapt to contour around roots, thus contributing to the ease of root coverage procedure by making it less technically demanding and significantly reducing the surgical time [18]. AM used with CAF demonstrated stable results at the 18-month follow-up [30]. Besides, AM can be used to provide a significant root coverage outcome, increase the thickness of keratinized gingiva, and improve gingival biotype [22,31]. It closely mimics the human mucosa basement membrane and contains laminin-5, which plays a role in the cellular adhesion of gingival cells. Other than the laminins, the basement membrane of AM contains collagen types III, IV, and V, and cell-adhesion bioactive factors including glycoproteins and fibronectins. The self-adhesive property of AM helps reduce operatory time because it does not require a second surgical site in the root coverage procedure [31].

Aesthetics

Gingival recession appears clinically as the display of the root surface of the tooth due to the displacement of the gingival margin apically from the cementoenamel junction and is thus associated with multiple aesthetics and functional problems such as exposed root, cervical/root caries, tooth hypersensitivity, and pulp hyperemia. Therefore, the ultimate goal of any root coverage procedure is complete and stable coverage of the recession defect. AM provides excellent aesthetic results in terms of texture and color match to the recipient site and results in a complete root coverage for gingival recession defects [22]. The subepithelial connective tissue grafts technique is considered the “gold standard” of root coverage procedures. Remarkably, Lafzi et al. [28] observed that AM with CAF is relatively comparable with the gold standard. In fact, satisfaction with aesthetic results of AM was higher. In a randomized clinical control study, AM was used as a biological dressing at the palatal donor site after harvesting the soft tissue graft [25]. The observation after 21 days showed excellent color match and tissue texture of the palatal donor site with the adjacent tissue [25]. Owing to its aesthetic properties, AM could be one of the considered options in oral cavity defect reconstruction procedures.

Improvement in Gingival Biotype

AM is used in conjunction with CAF in root coverage procedures in Miller’s class I and class II gingival recession defects to provide stable and significant root coverage and increase the thickness of keratinized gingiva [26,28,30-32]. It is not surprising to note that some results showed a complete (100%) if not near-complete root coverage since AM has many exceptional properties that make it a membrane of choice to be used with CAF as a combined approach in treating gingival recessions [28,31]. The root coverage was stable even after 18 months postoperatively by maintaining the structural and anatomical configuration of the regenerated tissues without any adverse effect. In another study, it was demonstrated that CAF with AM and PRF both achieved 100% root coverage and enhanced the gingival biotype in bilateral multiple Miller’s class I recession. Furthermore, the AM-treated sites demonstrated more stable results than the PRF-treated sites at the end of the seventh month [26]. This finding was consistent with that of Rehan et al. [30] who also compared the effectiveness of CAF with AM and PRF in the treatment of Miller’s class I recession defects in an 18-month study. The authors concluded that both membranes are equally effective in providing clinically significant outcomes with respect to root coverage in which AM shows a better percentage of root coverage as compared to PRF [30]. Because of its better stability and ease of handling, the application of AM as a novel approach to root coverage could be more desirable than PRF.

Potential Scaffold for Regeneration

Other than being used in a combined approach with CAF clinically, AM acts as a scaffold for periodontal ligament cell growth from in vitro studies [11,21]. The basement membrane of the AM contains extracellular matrix components that produce a nearly native scaffold for cell seeding, thus suitable to be applied in the periodontal regenerative procedure [8,42]. In the study of Iwasaki et al., PDLSC-transferred AM was found to have a therapeutic potential on periodontal tissue regeneration by significantly enhancing the formation of periodontal tissues in vivo [21]. Meanwhile, Elahi et al. observed that human periodontal ligament fibroblasts (HPDLFs) can attach, proliferate, and integrate with AM, which indicated that AM is biocompatible and can be a promising scaffold for periodontal regeneration [11].

AM and adipose-derived stem cell-co culture systems could increase bone regeneration in a periodontal osseous defect rat model by forming more hard tissues and showing better defect recovery [24]. Therefore, the combination of tissue engineering technology utilizing AM and stem cell therapy to regenerate periodontal bone is very encouraging in patients with periodontal disease who suffer from tooth loss. Nevertheless, as described earlier, AM is a very thin membrane and delicate, thus requiring proper handling during application [7,42]. Hence, having a thorough understanding of its physical characteristics as well as expert operators manipulating AM during periodontal regenerative procedures may aid in achieving good AM adaptation to the defect site.

## Conclusions

Based on this review, it is evidenced that AM has unique structure and components contributing to its exceptional properties such as anti-inflammatory, low immunogenicity, anti-scarring, antimicrobial, promoting epithelialization, reduction of pain, and improving gingival biotypes, as well as a suitable platform for periodontal cells growth. Owing to these various beneficial properties, AM may serve as a potential alternative natural biomaterial that can be used for regenerative periodontal therapy. However, more clinical trials are recommended to further elucidate its efficacy and sustainability to act as a scaffold in periodontal regeneration.
